# Sickle Cell Disease Presenting as Extradural Hematoma: An Extremely Rare Fatal Crisis

**DOI:** 10.7759/cureus.27004

**Published:** 2022-07-19

**Authors:** Divit Shah, Harshita Reddy, Sunil Kumar, Sourya Acharya

**Affiliations:** 1 Medicine, Jawaharlal Nehru Medical College, Datta Meghe Institute of Medical Sciences, Wardha, IND

**Keywords:** anaemia, haemorrhage., complication, hemolysis, sickle cell

## Abstract

Sickle cell disease (SCD) is due to a gene mutation in the hemoglobin subunit beta gene, whose polymerization when deoxygenated disrupts the physicochemical properties of red blood cells, triggering pan-cellular activation and pathogenic processes such as hemolysis, vaso-occlusion, and ischemia-reperfusion, culminating in the disease's numerous and severe complication like hemorrhage. This case report highlights spontaneous extradural hematoma in a young adult with SCD as an acute neurological complication.

## Introduction

Sickle cell disease (SCD) happens due to an alteration of the beta-globin chain (valine substituting glutamic acid), which increases the hemoglobin molecule's proclivity to polymerize in a deoxygenated state. This disease is more common in central and southern parts of India, the second most common hemoglobinopathy after thalassemia [1].

Neurologic manifestations as a result of SCD are prevalent, afflicting nearly 35% of individuals. Ischemic strokes (75%), intra-parenchymal hemorrhage (25%), and sub-arachnoid hemorrhage, sub-galeal hematoma, and extra-dural/epidural hematoma are the most often recorded cerebrovascular consequences of SCD [2,3]. Extradural hematoma has been reported in scrub typhus and Graves' disease. [3,4]

A complication of SCD called isolated non-traumatic spontaneous epidural hematoma (EDH) is extremely rare. Infectious diseases, coagulation disorders linked with end-stage renal disease and haemodialysis, dural metastases, and Langerhans cell histiocytosis have all been documented as causes of spontaneous EDH.

In this case report, we present a 19-year-old patient of sickle cell disease with an extremely rare neurological crisis as a fatal extradural hematoma.

## Case presentation

A 19-year-old male from a rural part of central India, in Wardha, was diagnosed with homozygous (HbSS) sickle cell disease at 12 years of age with a positive family history. During adolescence, he had multiple admissions for the painful vaso-occlusive crisis. This time, he presented to the outpatient medicine department with complaints of severe body ache and intermittent headache for 3 days. He was on symptomatic medical (analgesics in the form of ibuprofen 400 mg intermittently and hydration) treatment without hydroxyurea. In the past, he had multiple blood transfusions. He denied any history of smoking and alcohol intake. The patient had no prior co-morbidities like diabetes mellitus hypertension or tuberculosis. There was no history of gum bleeding or ecchymosis on the body.

The patient’s vitals on admission were pulse was 102 beats/minute with a regular rhythm, blood pressure 110/80 mmHg, respiratory rate 28/minute, with oxygen saturation of 96% on room air. Tachypnea was likely due to acute chest crisis, though ABG (arterial blood gas analysis) was normal. On general examination, he was having icterus and mild, non-tender splenomegaly measuring 16 cms. Other systemic examinations were within normal limits. There was no sternal or bony tenderness. All the laboratory investigations of the patients are listed in Table 1.

**Table 1:** Laboratory investigation on Day 1 and Day 3.

parameters	Day 1	Day 3
Haemoglobin	10.6 g/dL	10.0 g/dL
TLC (total leucocyte count)	12,100 cell/mm3	13,500 cell/mm3
Platelet count	0.7 lakh/mm3	0.68 lakh/mm3
Serum bilirubin	7.2 mg/dL	8.3 mg/dL
aspartate aminotransferase (AST)	145 IU/L	158 IU/L
alanine aminotransferase (ALT)	180 IU/L	178 IU/L
Serum albumin	3.2 mg/dL	3.1 mg/dL
Serum creatinine	1.5 mg/dL	1.4 mg/dL
Serum sodium	136 mmol/L	138 mmol/l
Serum potassium	3.4 mmol/L	4.0 mmol/l
INR (international normalised ratio)	1.2	1.1
Prothrombin time	11 seconds	10 seconds
activated partial thromboplastin time	22 seconds	21 seconds

**Figure 1:**
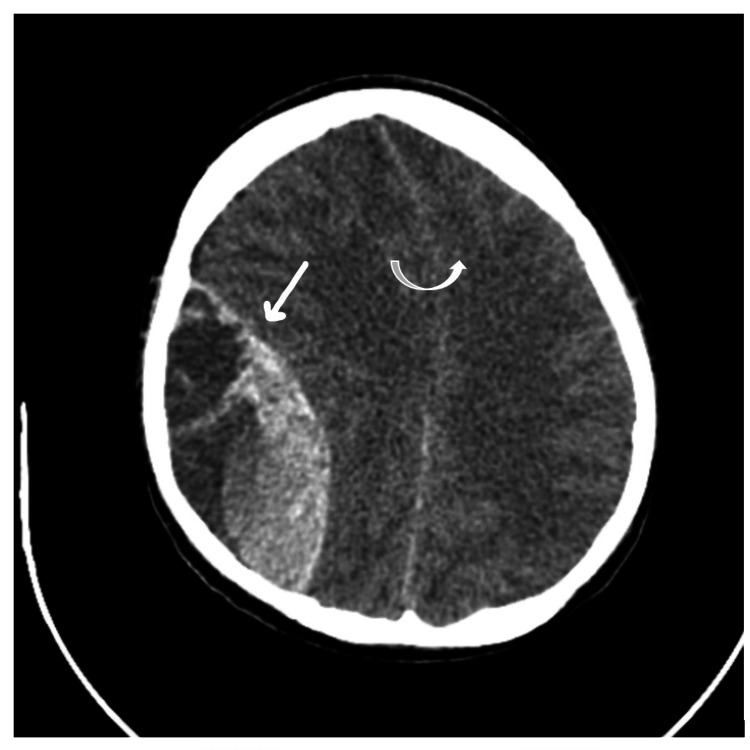
Right-sided extradural hematoma (straight arrow) and subfalcine herniation (curved arrow).

On day 3, the patient had a severe headache with altered sensorium for which computerized tomography (CT) scanning of the brain was planned which revealed a large extradural hematoma in the right parietal region with mass effect and subfalcine herniation (Figure 1). 

**Figure 2:**
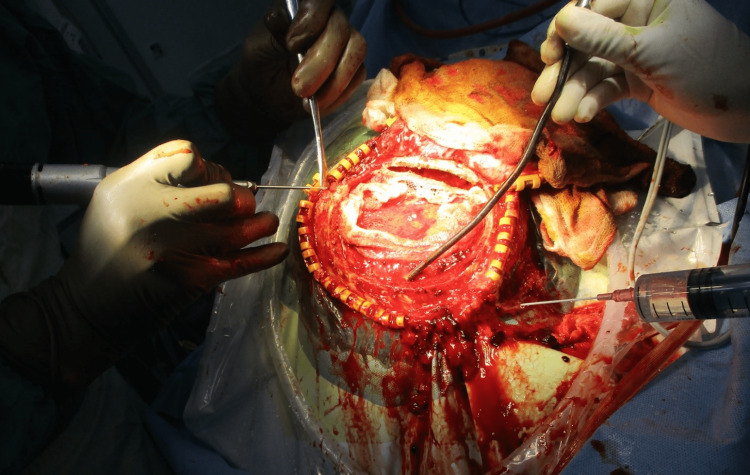
Intraoperative picture of hematoma removal.

In meantime, he became drowsy and his GCS (Glasgow Coma Scale) become 6. He was immediately intubated with an endotracheal tube size 7.5 mm and connected to the ventilator, and the opinion of a neurosurgical expert was taken. The patient was transfused a single unit of single-donor platelet and was taken for emergency decompression surgery (Figure 2).

**Figure 3:**
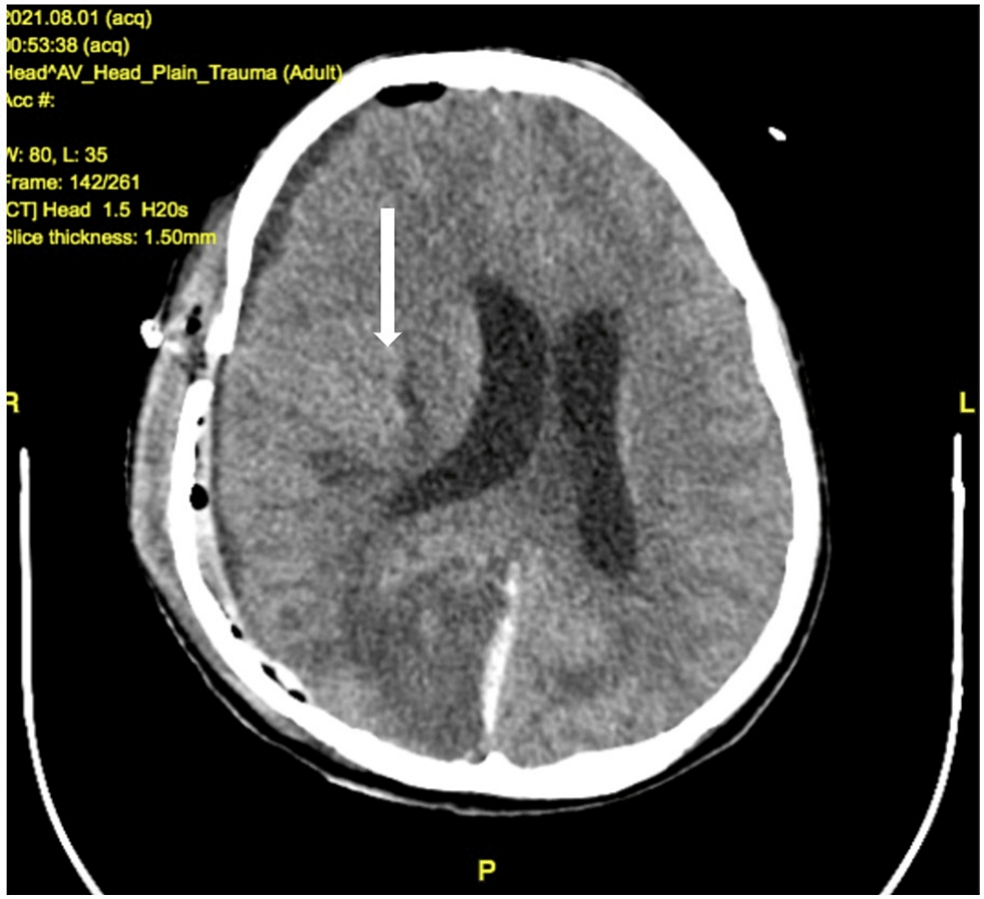
Postoperative CT brain showing diffuse cerebral edema with a significant decrease in the hematoma.

Postoperative CT-brain was done after 12 hours, which revealed a significant decrease in hematoma but diffuse cerebral edema, and in situ drain was placed subcutaneously as shown in Figure 3.

Postoperative, the patient was kept on ventilatory support, along with neuroprotective strategies, mannitol, and broad-spectrum antimicrobial drugs. However, the patient succumbed even after the best resuscitation.

## Discussion

Central nervous system problems are the most significant and morbid consequences of SCD [5]. Extradural hematomas, in addition to infarction and hemorrhagic strokes, can develop in children with SCD. However, there is a scarcity of research detailing the prevalence and consequences of extradural hemorrhagic episodes in SCD, particularly in young adults. Although EDH is uncommon, its long-term consequences can be severe, and the related death rate is significant. Hamm et al. reported seven children with SCD with cranial epidural hematomas having a mortality rate of 20% [6]. As a result, if EDH is suspected in an SCD patient, neuroimaging should be conducted as soon as possible.

A rare consequence of sickle cell illness is a spontaneous extradural hematoma. The formation of extradural hematomas in SCD has been linked to unestablished pathophysiologies like vaso-occlusion of the hematopoietic calvarial diploic bone resulting in bone infarction and blood leaking into the periosteal, epidural, and sub-galeal regions [2,7]. Acute and rapid increase in hematopoiesis, resulting in microfracture of the already thin inner cortex and blood and hematopoietic tissue extravasation with sludging of sickle cells in the diploic veins, obstructing venous drainage, and causing blood leaking due to vascular injury have also been postulated. [7] Coagulopathy or platelet dysfunction is also a common cause of hematoma development and poor clinical outcomes [2,7]. However, in our case, it remains obscured what led to the sudden extradural hematoma, which leads to fatality. Low platelet can be one of the possibilities in our case, which was 70,000/mm3, which may lead to bleeding. Abnormal platelet counts are relatively common, especially thrombocytopenia rather than thrombocytosis. In SCD patients with acute chest syndrome, platelet count <200 × 109/L was found to be an independent predictor of respiratory failure and neurologic complications [7].

## Conclusions

Although vascular crisis is very common in SCD, an uncommon complication/crisis of sickle cell disease is a spontaneous extradural hematoma. When individuals appear with a sudden headache, it should be suspected. Although rare, extradural hematoma can be fatal and should be considered in patients with acute neurological symptoms.
